# The impact of HIV infection on tuberculosis transmission in a country with low tuberculosis incidence: a national retrospective study using molecular epidemiology

**DOI:** 10.1186/s12916-020-01849-7

**Published:** 2020-12-14

**Authors:** Joanne R. Winter, Colette J. Smith, Jennifer A. Davidson, Maeve K. Lalor, Valerie Delpech, Ibrahim Abubakar, Helen R. Stagg

**Affiliations:** 1 Institute for Global Health, University College London, London, UK; 2 Tuberculosis Unit, National Infection Service, Public Health England, London, UK; 3 HIV Unit, National Infection Service, Public Health England, London, UK; 4 Usher Institute, University of Edinburgh, Edinburgh, UK

**Keywords:** Tuberculosis, HIV, Co-infection, Transmission, MIRU-VNTR

## Abstract

**Background:**

HIV is known to increase the likelihood of reactivation of latent tuberculosis to active TB disease; however, its impact on tuberculosis infectiousness and consequent transmission is unclear, particularly in low-incidence settings.

**Methods:**

National surveillance data from England, Wales and Northern Ireland on tuberculosis cases in adults from 2010 to 2014, strain typed using 24-locus mycobacterial-interspersed-repetitive-units–variable-number-tandem-repeats was used retrospectively to identify clusters of tuberculosis cases, subdivided into ‘first’ and ‘subsequent’ cases.

Firstly, we used zero-inflated Poisson regression models to examine the association between HIV status and the number of subsequent clustered cases (a surrogate for tuberculosis infectiousness) in a strain type cluster. Secondly, we used logistic regression to examine the association between HIV status and the likelihood of being a subsequent case in a cluster (a surrogate for recent acquisition of tuberculosis infection) compared to the first case or a non-clustered case (a surrogate for reactivation of latent infection).

**Results:**

We included 18,864 strain-typed cases, 2238 were the first cases of clusters and 8471 were subsequent cases. Seven hundred and fifty-nine (4%) were HIV-positive.

Outcome 1: HIV-positive pulmonary tuberculosis cases who were the first in a cluster had fewer subsequent cases associated with them (mean 0.6, multivariable incidence rate ratio [IRR] 0.75 [0.65–0.86]) than those HIV-negative (mean 1.1).

Extra-pulmonary tuberculosis (EPTB) cases with HIV were less likely to be the first case in a cluster compared to HIV-negative EPTB cases. EPTB cases who were the first case had a higher mean number of subsequent cases (mean 2.5, IRR (3.62 [3.12–4.19]) than those HIV-negative (mean 0.6).

Outcome 2: tuberculosis cases with HIV co-infection were less likely to be a subsequent case in a cluster (odds ratio 0.82 [0.69–0.98]), compared to being the first or a non-clustered case.

**Conclusions:**

Outcome 1: pulmonary tuberculosis-HIV patients were less infectious than those without HIV. EPTB patients with HIV who were the first case in a cluster had a higher number of subsequent cases and thus may be markers of other undetected cases, discoverable by contact investigations.

Outcome 2: tuberculosis in HIV-positive individuals was more likely due to reactivation than recent infection, compared to those who were HIV-negative.

**Supplementary Information:**

The online version contains supplementary material available at 10.1186/s12916-020-01849-7.

## Background

HIV infection increases susceptibility to tuberculosis (TB) disease by increasing the rate of progression from latent TB infection (LTBI) to active disease [1, 2]. However, there is also evidence that overall, TB may be less infectious in patients who also have HIV; contact studies have shown lower prevalence of tuberculin skin test (TST) positivity and lower TST conversion rates among contacts of HIV-positive index patients than HIV-negative index patients [3–5], particularly when index patients with HIV were immunocompromised [6]. This may be mediated through a shorter duration of infectiousness due to accelerated TB disease progression resulting in earlier diagnosis [2, 7], earlier TB treatment [6], lower rates of cavitary [4, 6] or sputum smear-positive [4, 5] TB, or a shorter duration of cough [4] among HIV-positive index patients.

Molecular strain typing data can help identify cases which may be part of the same chain of transmission [8]. Since 2010, all culture-positive *Mycobacterium tuberculosis* complex (*MTBC*) isolates in England, Wales and Northern Ireland have been prospectively strain typed using 24-locus mycobacterial interspersed repetitive units–variable number tandem repeats (MIRU-VNTR) typing. 58.4% of TB cases in England were part of a strain type cluster with at least one other case between 2010 and 2015 [9, 10].

Several studies in low-incidence settings which examined whether HIV was a risk factor for being part of a strain type cluster found no association [11–13], including one meta-analysis [14], but other more recent studies have reported both positive [15] and negative [16, 17] associations. Weak evidence from studies in low-burden settings (with few HIV-positive TB cases) suggests that HIV positivity among the first cases of a cluster may be associated with increased numbers of secondary cases in clusters (possibly because contacts of HIV-infected TB patients may be more likely to have HIV themselves, and therefore may be more susceptible to TB infection) and that patients with TB arising from recent infection are more likely to be HIV-positive than patients whose TB derives from reactivation of LTBI [18–20]. Larger cluster sizes in these studies were also associated with social risk factors such as illicit/intravenous drug use and homelessness, both of which are commonly associated with HIV co-infection.

Most risk factors for TB transmission have the same direction of effect on both susceptibility to infection and likelihood of onward transmission. In contrast, HIV may increase susceptibility to infection and is known to increase progression to active TB disease, but may lower infectiousness of TB. The overall impact of HIV on onward transmission of TB is therefore unclear, particularly in low-incidence settings. We utilised a comprehensive national dataset of TB notifications over 5 years, combined with molecular strain typing data and linked to national HIV surveillance data, to examine two outcomes. Firstly, we examined whether the HIV status of a TB case determined the number of subsequent clustered cases. Secondly, we assessed whether TB is more often due to reactivation of LTBI or recent infection in patients with and without HIV.

## Methods

### Study population

This was a retrospective study of culture-confirmed patients with *MTBC* disease in adults (aged ≥ 15 years) in England, Wales and Northern Ireland, notified to Public Health England (PHE)‘s Enhanced TB Surveillance System (ETS) between 2010 and 2014. We included all notified TB patients whose *MTBC* isolates were strain typed at ≥ 23 loci, using 24-loci MIRU-VNTR genotyping [8]. Recurrent TB cases were identified by record linkage and excluded if the strain type of recurrent notifications was indistinguishable from that of the first (i.e. plausible instances of relapse of active TB disease).

### Defining strain type clusters

PHE defines a strain type cluster as two or more persons with TB caused by indistinguishable MIRU-VNTR strain types [8, 21]. TB cases with unique strain types were considered ‘not clustered’.

The earliest date of evidence of TB disease for each patient (including symptom onset date, date of presentation to healthcare, earliest specimen date, diagnosis date, treatment start date and case notification date) was used to define the order of cases within clusters. We defined the earliest patient in each cluster as the first case and all later cases as subsequent cases.

Cases of TB in children (aged < 15 years) were included in the dataset when determining the order of TB cases within a cluster. However, as HIV status could only be determined for adults, we excluded children from our subsequent analyses. As TB is rare in the UK, clusters were not limited by geographical area within England, Wales and Northern Ireland.

### Statistical analysis

Data were analysed in Stata version 13.1. Descriptive analyses of the cohort were undertaken, examining the proportion of cases belonging to a strain type cluster and how many of whom were first cases compared to subsequent cases, stratified by HIV status. We also examined the number of subsequent cases following the first case of pulmonary TB in a cluster, stratified by HIV status of the first case in the cluster.

To investigate whether HIV was a risk factor for potential transmission of TB, we conducted two analyses, described in detail below.

#### Outcome 1: Likelihood of transmitting TB, and the number of subsequent TB cases

This analysis aimed to assess whether the HIV status of a TB case affected transmission, determined by the number of subsequent clustered cases. We compared the likelihood of transmission from TB cases with unique strain types versus those who were the first case in a cluster. The number of subsequent cases for the first case of a cluster was calculated as the number of patients in the cluster, minus one. TB cases with unique strain types were classed as having zero subsequent cases.

To investigate the impact of HIV on the onward transmission of TB, multivariable zero-inflated Poisson regression [22] was used to examine whether the HIV status of the first case of a cluster determined the number of subsequent clustered cases.

Zero-inflated Poisson regression is useful for modelling count data with an excess of zeroes, when the underlying theory suggests that the excess zeroes occur due to a separate process, and can therefore be modelled separately. In this study, we suggest that TB patients fall into two groups; those who are not infectious (and therefore cannot transmit TB to anyone else), modelled by a logistic model, and those who are infectious (and may therefore transmit TB to none, one, or more people), modelled by a Poisson model. Zero-inflated Poisson regression models undertake both of these processes and therefore give an output in two parts: an odds ratio (for the odds of transmitting infection to any subsequent patients) and a rate ratio (for the number of subsequent clustered cases, given that there has been transmission of infection). The model was offset by the time since the earliest date of evidence of TB to the end of the study period (31 December 2014). This analysis was subdivided by the site of TB disease of the first case in the cluster (pulmonary disease with or without extra-pulmonary disease, compared to extra-pulmonary disease only), as it is generally accepted that patients with only extra-pulmonary TB (EPTB) are not infectious, and adjusted for other confounding variables [23].

As the first identified case of the cluster may not be responsible for transmission within the cluster, we conducted a sensitivity analysis in which we examined the number of subsequent cases for the first pulmonary case in each cluster, regardless of whether the first pulmonary case was the first case in the cluster.

#### Outcome 2: Likelihood of being a subsequent case in a cluster (a surrogate for recent TB infection)

This analysis investigated whether HIV status influenced whether a patient’s TB was more likely to be the result of recent infection or reactivation of LTBI. We used multivariable logistic regression to assess the odds ratio for being a subsequent case in a cluster (a proxy for recent acquisition of TB infection), compared to being the first case or a non-clustered case (representing reactivation cases) in HIV-positive and negative individuals. All TB cases with strain typing data were included in this analysis.

As per outcome 1, we also conducted a sensitivity analysis in which we assumed that transmission originated from the first pulmonary case in the cluster, rather than the first case temporally irrespective of the site of disease.

### Exposure variables

Our primary exposure variable was HIV status, which was determined through linkage [24, 25] of ETS to the national HIV and AIDS Reporting System [26, 27]. Potential confounders for the relationship between HIV status and the outcomes were identified prospectively [23, 28] and are shown in Table 1*.* All potential confounders were included in the multivariable models.

**Table 1:** The clustering status of TB cases by risk factor in England, Wales and Northern Ireland, 2010–2014

	Total cases	Clustered cases (%)	Subsequent cases (% of clustered cases)	First cases (% of clustered cases)
**HIV status**				
Negative	18,105	10,299 (56.9)	8160 (79.2)	2139 (20.8)
Positive	759	410 (54.0)	311 (75.9)	99 (24.1)
**Year of TB notification**				
2010	3174	1795 (56.6)	874 (48.7)	921 (51.3)
2011	4296	2443 (56.9)	1786 (73.1)	657 (26.9)
2012	4327	2525 (58.4)	2150 (85.1)	375 (14.9)
2013	3696	2130 (57.6)	1940 (91.1)	190 (8.9)
2014	3371	1816 (53.9)	1721 (94.8)	95 (5.2)
**Sex**				
Female	7521	4153 (55.2)	3272 (78.8)	881 (21.2)
Male	11,323	6547 (57.8)	5196 (79.4)	1351 (20.6)
Missing	20	9 (45.0)	3 (33.3)	6 (66.7)
**Age (years)**				
15–24	3238	2059 (63.6)	1652 (80.2)	407 (19.8)
25–34	5632	3139 (55.7)	2453 (78.1)	686 (21.9)
35–44	3578	2041 (57.0)	1601 (78.4)	440 (21.6)
45–54	2388	1423 (59.6)	1149 (80.7)	274 (19.3)
55–64	1488	890 (59.8)	717 (80.6)	173 (19.4)
65+	2540	1157 (45.6)	899 (77.7)	258 (22.3)
**Ethnicity**				
White	3991	2442 (61.2)	1959 (80.2)	483 (19.8)
Black African	3211	2031 (63.3)	1603 (78.9)	428 (21.1)
Black Other	588	458 (77.9)	391 (85.4)	67 (14.6)
Indian sub-continent	8079	4198 (52.0)	3300 (78.6)	898 (21.4)
Mixed/other	2525	1330 (52.7)	1029 (77.4)	301 (22.6)
Missing	470	250 (53.2)	189 (75.6)	61 (24.4)
**Time since entry to the UK**				
UK born	4431	3000 (67.7)	2495 (83.2)	505 (16.8)
Within 2 years	2535	1313 (51.8)	979 (74.6)	334 (25.4)
2–5 years	2999	1509 (50.3)	1154 (76.5)	355 (23.5)
5–10 years	2743	1485 (54.1)	1149 (77.4)	336 (22.6)
More than 10 years	4115	2329 (56.6)	1870 (80.3)	459 (19.7)
Missing	2041	1073 (52.6)	824 (76.8)	249 (23.2)
**TB lineage**				
Beijing	1041	770 (74.0)	667 (86.6)	103 (13.4)
Euro-American	7313	4300 (58.8)	3352 (78.0)	948 (22.0)
Central Asian Strain	5280	3285 (62.2)	2674 (81.4)	611 (18.6)
East Asian Indian	2674	1046 (39.1)	769 (73.5)	277 (26.5)
Other/unknown	2554	1306 (51.1)	1008 (77.2)	298 (22.8)
Missing	2			
**IMD decile**				
1	3933	2360 (60.0)	1868 (79.2)	492 (20.8)
2	3645	2130 (58.4)	1678 (78.8)	452 (21.2)
3	3008	1704 (56.6)	1334 (78.3)	370 (21.7)
4	2301	1314 (57.1)	1066 (81.1)	248 (18.9)
5	1655	906 (54.7)	695 (76.7)	211 (23.3)
6	1183	652 (55.1)	516 (79.1)	136 (20.9)
7	838	453 (54.1)	375 (82.8)	78 (17.2)
8	728	398 (54.7)	302 (75.9)	96 (24.1)
9	610	307 (50.3)	241 (78.5)	66 (21.5)
10	474	243 (51.3)	194 (79.8)	49 (20.2)
Missing	489	242 (49.5)	202 (83.5)	40 (16.5)
**Drug misuse**				
No	16,536	9241 (55.9)	7291 (78.9)	1950 (21.1)
Yes	702	551 (78.5)	473 (85.8)	78 (14.2)
Missing	1626	917 (56.4)	707 (77.1)	210 (22.9)
**Alcohol misuse**				
No	16,260	9160 (56.3)	7251 (79.2)	1909 (20.8)
Yes	776	528 (68.0)	441 (83.5)	87 (16.5)
Missing	1828	1021 (55.9)	779 (76.3)	242 (23.7)
**Homelessness**				
No	16,771	9480 (56.5)	7500 (79.1)	1980 (20.9)
Yes	666	449 (67.4)	372 (82.9)	77 (17.1)
Missing	1427	780 (54.7)	599 (76.8)	181 (23.2)
**Imprisonment**				
No	16,210	9097 (56.1)	7200 (79.1)	1897 (20.9)
Yes	649	484 (74.6)	410 (84.7)	74 (15.3)
Missing	2005	1128 (56.3)	861 (76.3)	267 (23.7)
**Site of TB disease/smear status**^**†**^				
Pulmonary, smear positive	4959	3137 (63.3)	2448 (78.0)	689 (22.0)
Pulmonary, smear negative/unknown	6952	4084 (58.7)	3279 (80.3)	805 (19.7)
Extra-pulmonary	6947	3486 (50.2)	2742 (78.7)	744 (21.3)
Missing	6	2 (33.3)	2 (100.0)	0 (0.0)

*IMD:* index of multiple deprivation score. IMD score deciles represent relative levels of deprivation of income, employment, health, education, housing and services, crime and living environment for small areas in England and Wales, where 1 = most deprived and 10 = least deprived [29, 30]

^**†**^Patients with both pulmonary and extra-pulmonary disease were classed as having pulmonary disease

## Results

### Descriptive analysis

**Fig. 1:**
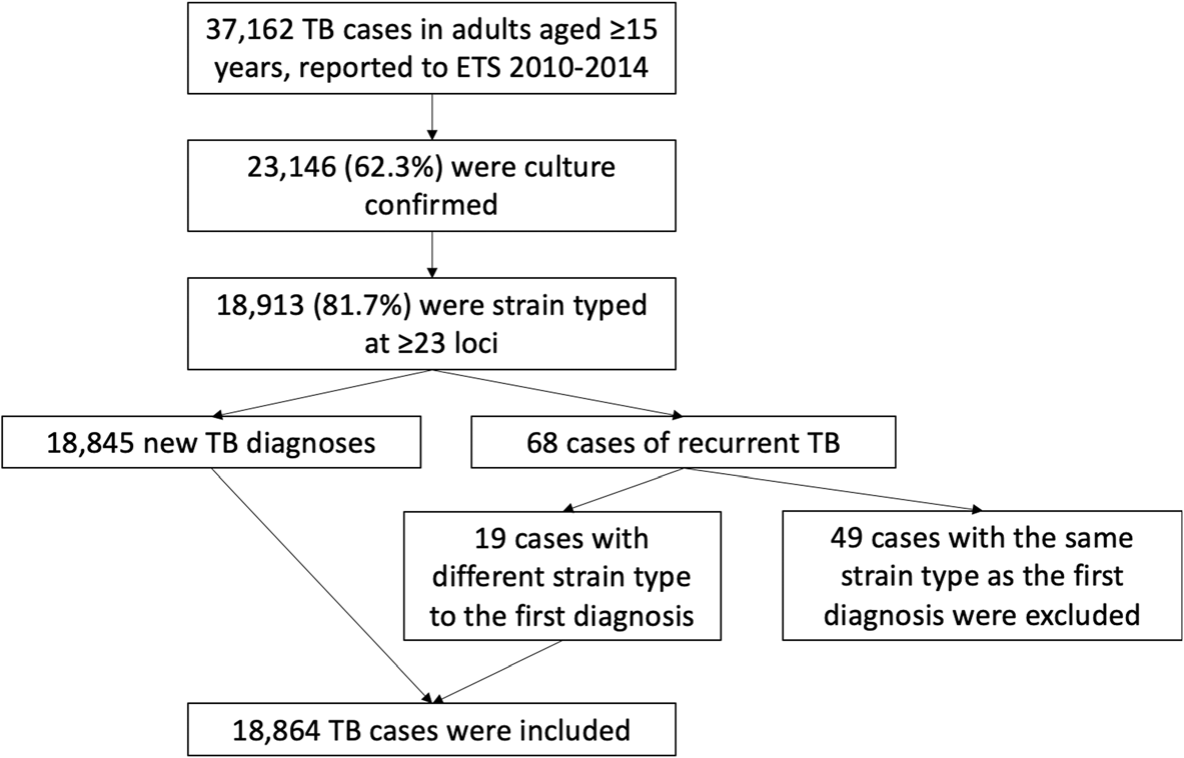
Flow chart of included cases

A flow chart of the cases included is shown in Fig. 1. 37,162 cases of TB in adults aged ≥ 15 years were notified to PHE in England, Wales and Northern Ireland between 2010 and 2014. 23,146 (62.3%) were culture confirmed, of which 18,913 (81.7%) were strain typed at ≥ 23 loci. We excluded 49 cases of recurrent TB with the same strain type as the original infection; 19 recurrent instances of disease with different strain types were included. 18,864 TB cases were included in our analysis, representing 50.8% of TB cases in England, Wales and Northern Ireland from 2010 to 2014. Of the cases included in the analysis, 10,709 (56.8%) were part of 2284 strain type clusters. In total, 2238 (20.9%) were the first cases in a cluster (in 46 clusters the first case was aged < 15 years and therefore excluded from the statistical analysis) and 8471 (79.1%) were subsequent cases.

Seven hundred and fifty-nine TB cases were co-infected with HIV (4.0%); 410/759 (54.0%) were clustered and 99/410 (24.2%) were the first case in a cluster.

Of the 8471 subsequent cases in clusters, 3.7% were HIV-positive. 572/8471 (6.8%) of subsequent cases had an HIV-positive first case, 7775 (91.8%) had an HIV-negative first case, and the HIV status of the first case was unknown for 124 (1.5%) patients from clusters in which the first case was a child. Other demographic, socioeconomic and clinical factors are shown in Table 1.

The HIV status of the first case of a cluster was positively associated with the HIV status of subsequent cases (*χ*^2^ test *P* < 0.001). The prevalence of HIV among subsequent cases was higher in clusters with an HIV-positive first case (10.7%) than in clusters with an HIV-negative first case (3.2%). 6.4% of HIV-negative subsequent cases had an HIV-positive first case, compared to 19.9% of HIV-positive subsequent cases. 1998/2284 (87.5%) of clusters consisted of only HIV-negative TB patients, 11 clusters (0.5%) consisted of only HIV-positive TB patients and 275 (12.0%) clusters were mixed.

The mean cluster size in the cohort was 5 (median 3, inter-quartile range 2–4, range 2–198), 5 for clusters where the first patient was HIV-negative and 7 for clusters with an HIV-positive first case.

#### Outcome 1: The impact of HIV on the likelihood of transmitting TB, and the number of subsequent TB cases

The number of subsequent cases following the first TB case in a cluster differed substantially by HIV status, site of disease and smear status (Table 2).

**Table 2:** The mean number of subsequent clustered cases, stratified by the HIV status, site of disease and smear status of the first case

Site of disease^**†**^ and smear status	HIV status of first case		
	HIV-negative Mean (SE)	HIV-positive Mean (SE)	Total Mean (SE)
Pulmonary smear positive	1.1 (0.02)	0.6 (0.07)	1.1 (0.02)
Pulmonary smear negative/unknown	0.8 (0.01)	0.9 (0.07)	0.8 (0.01)
Extra-pulmonary disease	0.6 (0.01)	2.5 (0.14)	0.7 (0.01)
Total	0.8 (0.01)	1.3 (0.05)	0.8 (0.01)

*Mean:* arithmetic mean. *SE:* standard error of the mean (Poisson distribution)

^**†**^Patients with both pulmonary and extra-pulmonary disease were classed as having pulmonary disease

The zero-inflated Poisson model showed that among pulmonary TB cases (with or without extra-pulmonary disease), there was no evidence for an association between HIV co-infection and being the first case of a strain type cluster (compared to not being part of a strain type cluster) in the logistic part of the model (multivariable odds ratio [OR] 1.10 [0.79–1.53], Table 3). However, HIV co-infection was associated with a decreased number of subsequent clustered cases in the Poisson part of the models (multivariable incidence rate ratio [IRR] 0.75 [0.65–0.86], Table 3). This shows where TB cases with HIV were the first case of a cluster, the overall cluster size was smaller.

**Table 3:** Univariable and multivariable zero-inflated Poisson regression of factors associated with the likelihood of transmitting TB, and the number of subsequent clustered cases for pulmonary TB cases in England, Wales and Northern Ireland, 2010–2014

	Total pulmonary cases	Clustered pulmonary cases (%)	First pulmonary cases (% of clustered cases)	Univariable (number of subsequent cases)	Univariable (non-clustered case)	Multivariable^**≠**^ (number of subsequent cases)	Multivariable^**≠**^ (non-clustered case)
				IRR (95% CI)	OR (95% CI)	IRR (95% CI)	OR (95% CI)
**HIV status**							
Negative	11,366	6910 (60.8)	1950 (28.2)	1.00	1.00	1.00	1.00
Positive	545	311 (57.1)	106 (34.1)	0.76 (0.68–0.87)	0.94 (0.72–1.23)	0.75 (0.65–0.86)	1.10 (0.79–1.53)
**Year of TB diagnosis**							
2010	2028	1205 (59.4)	716 (59.4)	1.00	1.00	1.00	1.00
2011	2696	1638 (60.8)	546 (33.3)	0.63 (0.59–0.66)	1.69 (1.46–1.96)	0.64 (0.60–0.68)	1.52 (1.29–1.80)
2012	2650	1670 (63.0)	379 (22.7)	0.39 (0.35–0.43)	1.87 (1.56–2.24)	0.38 (0.34–0.43)	1.53 (1.25–1.88)
2013	2354	1456 (61.9)	230 (15.8)	0.42 (0.35–0.49)	2.79 (2.20–3.53)	0.40 (0.34–0.48)	2.38 (1.83–3.11)
2014	2183	1252 (57.4)	185 (14.8)	0.64 (0.52–0.79)	4.53 (3.34–6.14)	0.59 (0.47–0.74)	4.04 (2.87–5.69)
**Sex**							
Female	4562	2661 (58.3)	765 (28.7)	1.00	1.00	1.00	1.00
Male	7333	4552 (62.1)	1285 (28.2)	1.04 (0.99–1.09)	0.86 (0.76–0.96)	1.01 (0.95–1.06)	0.81 (0.70–0.93)
Missing	16	8 (50.0)	6 (75.0)				
**Age (years)**							
15–24	2254	1504 (66.7)	405 (26.9)	0.93 (0.87–1.00)	0.78 (0.65–0.92)	0.86 (0.79–0.93)	0.78 (0.63–0.96)
25–34	3250	1947 (59.9)	575 (29.5)	1.00	1.00	1.00	1.00
35–44	2089	1314 (62.9)	395 (30.1)	1.30 (1.22–1.39)	0.89 (0.75–1.05)	1.33 (1.24–1.43)	0.99 (0.81–1.22)
45–54	1566	1000 (63.9)	252 (25.2)	0.90 (0.83–0.99)	0.89 (0.73–1.08)	0.96 (0.87–1.07)	1.08 (0.84–1.38)
55–64	999	633 (63.4)	167 (26.4)	1.19 (1.09–1.30)	1.10 (0.87–1.39)	1.01 (0.91–1.13)	1.36 (1.01–1.82)
65+	1753	823 (46.9)	262 (31.8)	1.04 (0.96–1.13)	1.61 (1.34–1.94)	1.03 (0.93–1.14)	1.97 (1.53–2.53)
**Ethnicity**							
White	3481	2205 (63.3)	522 (23.7)	1.00	1.00	1.00	1.00
Black African	1926	1270 (65.9)	370 (29.1)	0.96 (0.89–1.02)	0.76 (0.64–0.91)	1.23 (1.12–1.36)	0.91 (0.70–1.19)
Black Other	406	322 (79.3)	68 (21.1)	0.89 (0.77–1.02)	0.51 (0.35–0.74)	0.86 (0.74–1.01)	0.58 (0.37–0.93)
Indian sub-continent	4174	2354 (56.4)	758 (32.2)	0.94 (0.88–0.99)	1.15 (1.00–1.33)	0.93 (0.85–1.01)	1.19 (0.94–1.51)
Mixed/other	1621	894 (55.2)	273 (30.5)	0.60 (0.55–0.66)	1.03 (0.85–1.26)	0.68 (0.60–0.77)	1.10 (0.83–1.46)
Missing	303	176 (58.1)	65 (36.9)				
**Time since entry to the UK**							
UK born	3631	2526 (69.6)	540 (21.4)	1.00	1.00	1.00	1.00
Within 2 years	1536	833 (54.2)	311 (37.3)	0.70 (0.64–0.75)	1.10 (0.91–1.32)	0.65 (0.59–0.71)	1.27 (0.99–1.63)
2–5 years	1549	815 (52.6)	267 (32.8)	0.75 (0.69–0.81)	1.24 (1.02–1.50)	0.76 (0.69–0.84)	1.35 (1.05–1.74)
5–10 years	1543	897 (58.1)	283 (31.5)	0.82 (0.76–0.89)	1.10 (0.91–1.33)	0.76 (0.69–0.83)	1.25 (0.97–1.60)
More than 10 years	2423	1460 (60.3)	423 (29.0)	0.89 (0.83–0.95)	1.23 (1.04–1.46)	0.84 (0.77–0.91)	1.10 (0.87–1.40)
Missing	1229	690 (56.1)	232 (33.6)				
**TB lineage**							
Beijing	706	525 (74.4)	93 (17.7)	1.00	1.00	1.00	1.00
Euro-American	5306	3233 (60.9)	898 (27.8)	0.51 (0.47–0.56)	1.05 (0.80–1.39)	0.46 (0.41–0.50)	1.11 (0.80–1.54)
Central Asian Strain	2955	1948 (65.9)	547 (28.1)	0.72 (0.66–0.79)	1.05 (0.79–1.40)	0.78 (0.70–0.86)	1.01 (0.72–1.43)
East Asian Indian	1271	551 (43.4)	235 (42.6)	0.42 (0.37–0.48)	1.59 (1.16–2.17)	0.52 (0.45–0.59)	1.54 (1.06–2.23)
Other/unknown	1673	964 (57.6)	283 (29.4)	0.48 (0.43–0.53)	1.17 (0.86–1.59)	0.43 (0.38–0.48)	1.18 (0.82–1.69)
Missing	2						
**IMD decile**							
1	2581	1654 (64.1)	440 (26.6)	–	–	–	–
2	2238	1383 (61.8)	396 (28.6)	–	–	–	–
3	1851	1117 (60.3)	335 (30.0)	–	–	–	–
4	1425	873 (61.3)	247 (28.3)	–	–	–	–
5	1039	609 (58.6)	191 (31.4)	–	–	–	–
6	737	437 (59.3)	125 (28.6)	–	–	–	–
7	525	306 (58.3)	80 (26.1)	–	–	–	–
8	486	276 (56.8)	82 (29.7)	–	–	–	–
9	390	224 (57.4)	63 (28.1)	–	–	–	–
10	305	171 (56.1)	54 (31.6)	–	–	–	–
Missing	334	171 (51.2)	43 (25.1)	–	–	–	–
For each decile increase	–	–	–	0.96 (0.95–0.97)	1.02 (0.99–1.04)	0.96 (0.95–0.97)	1.00 (0.97–1.03)
**Drug misuse**							
No	10,165	6061 (59.6)	1768 (29.2)	1.00	1.00	1.00	1.00
Yes	639	507 (79.3)	82 (16.2)	1.14 (1.02–1.27)	0.61 (0.45–0.83)	0.88 (0.76–1.01)	0.84 (0.56–1.28)
Missing	1107	653 (59.0)	206 (31.5)				
**Alcohol misuse**							
No	10,039	6043 (60.2)	1747 (28.9)	1.00	1.00	1.00	1.00
Yes	670	470 (70.1)	87 (18.5)	1.85 (1.71–2.01)	0.97 (0.74–1.26)	1.69 (1.54–1.86)	1.18 (0.84–1.66)
Missing	1202	708 (58.9)	222 (31.4)				
**Homelessness**							
No	10,398	6277 (60.4)	1799 (28.7)	1.00	1.00	1.00	1.00
Yes	567	393 (69.3)	85 (21.6)	0.90 (0.80–1.02)	0.74 (0.55–0.99)	0.63 (0.54–0.72)	0.88 (0.59–1.30)
Missing	946	551 (58.2)	172 (31.2)				
**Imprisonment**							
No	9990	5978 (59.8)	1725 (28.9)	1.00	1.00	1.00	1.00
Yes	553	423 (76.5)	82 (19.4)	1.07 (0.96–1.20)	0.86 (0.76–0.96)	1.10 (0.97–1.26)	0.85 (0.57–1.26)
Missing	1368	820 (59.9)	249 (30.4)				
**Smear status**							
Smear positive	4959	3137 (63.3)	901 (28.7)	1.00	1.29	1.00	1.00
Smear negative or unknown	6952	4084 (58.7)	1155 (28.3)	0.87 (0.83–0.92)	1.94 (1.78–2.12)	0.83 (0.79–0.88)	1.17 (1.02–1.34)

*IRR:* incidence rate ratio (Poisson part) for an increased number of subsequent clustered cases. *OR:* odds ratio (zero-inflated part) for the odds of being a non-clustered case, compared to being the first case of a cluster. Both analyses were restricted to clusters where the first case was pulmonary. *IMD:* index of multiple deprivation score. IMD score deciles represent relative levels of deprivation of income, employment, health, education, housing and services, crime and living environment for small areas in England and Wales, where 1 = most deprived and 10 = least deprived [29, 30]

^≠^Adjusted for all variables shown in the table. The multivariable model included 5694 TB cases after 1052 were excluded due to missing data on one or more of sex (*n* = 14), ethnicity (*n* = 192), time since entry to the UK (*n* = 771) or IMD score (*n* = 206)

^**†**^Cases missing data were considered not to have these social risk factors

Extra-pulmonary (with no pulmonary disease) TB cases with HIV co-infection were less likely to be the first case of a cluster than those without HIV (multivariable OR for having a unique strain type 1.93 [1.12–3.33], Table 4). However, where an EPTB case was the first case in a cluster, HIV co-infection was associated with an increased number of subsequent cases (multivariable IRR 3.62 [3.12–4.19]).

**Table 4:** Univariable and multivariable zero-inflated Poisson regression of factors associated with the likelihood of being the first case of a cluster, and the number of subsequent clustered cases for extra-pulmonary TB cases in England, Wales and Northern Ireland, 2010–2014

	Total extra-pulmonary cases	Clustered cases (%)	First extra-pulmonary cases (% of clustered cases)	Univariable (number of subsequent cases)	Univariable (non-clustered case)	Multivariable^**≠**^ (number of subsequent cases)	Multivariable^**≠**^ (non-clustered case)
				IRR (95% CI)	OR (95% CI)	IRR (95% CI)	OR (95% CI)
**HIV status**							
Negative	6739	3389 (50.3)	722 (21.3)	1.00	1.00	1.00	1.00
Positive	214	99 (46.3)	22 (22.2)	4.16 (3.71–4.67)	1.38 (0.86–2.19)	3.62 (3.12–4.19)	1.93 (1.12–3.33)
**Year of TB diagnosis**							
2010	1146	590 (51.5)	293 (49.7)	1.00	1.00	1.00	1.00
2011	1600	805 (50.3)	242 (30.1)	0.77 (0.71–0.84)	1.65 (1.34–2.02)	0.72 (0.66–0.80)	1.45 (1.15–1.84)
2012	1677	855 (51.0)	122 (14.3)	0.56 (0.48–0.66)	2.84 (2.21–3.64)	0.60 (0.51–0.71)	2.57 (1.93–3.41)
2013	1342	674 (50.2)	62 (9.2)	0.39 (0.29–0.51)	2.83 (1.94–4.13)	0.45 (0.34–0.61)	2.82 (1.88–4.22)
2014	1188	564 (47.5)	25 (4.4)	0.90 (0.74–1.10)	6.86 (4.36–10.80)	1.11 (0.82–1.51)	7.82 (4.67–13.11)
**Sex**							
Female	2959	1492 (50.4)	323 (21.6)	1.00	1.00	1.00	1.00
Male	3990	1995 (50.0)	421 (21.1)	1.25 (1.15–1.35)	1.11 (0.94–1.31)	1.22 (1.12–1.34)	0.99 (0.81–1.21)
Missing	4	1 (25.0)	(0.0)				
**Age (years)**							
15–24	984	555 (56.4)	111 (20.0)	2.26 (2.01–2.54)	1.10 (0.85–1.42)	1.66 (1.46–1.89)	1.07 (0.80–1.45)
25–34	2382	1192 (50.0)	266 (22.3)	1.00	1.00	1.00	1.00
35–44	1489	727 (48.8)	156 (21.5)	1.67 (1.49–1.87)	1.27 (1.01–1.60)	1.43 (1.26–1.61)	1.32 (1.00–1.75)
45–54	822	423 (51.5)	83 (19.6)	1.37 (1.19–1.59)	1.19 (0.89–1.58)	1.39 (1.18–1.63)	1.46 (1.02–2.10)
55–64	489	257 (52.6)	52 (20.2)	1.73 (1.48–2.02)	1.18 (0.84–1.66)	1.92 (1.60–2.31)	1.45 (0.94–2.24)
65+	787	334 (42.4)	76 (22.8)	1.08 (0.92–1.26)	1.34 (1.00–1.81)	0.94 (0.78–1.14)	1.40 (0.92–2.12)
**Ethnicity**							
White	510	237 (46.5)	49 (20.7)	1.00	1.00	1.00	1.00
Black African	1285	761 (59.2)	150 (19.7)	1.76 (1.45–2.14)	0.74 (0.51–1.08)	0.85 (0.65–1.10)	0.49 (0.27–0.89)
Black Other	182	136 (74.7)	17 (12.5)	3.69 (2.92–4.66)	0.62 (0.32–1.19)	2.84 (2.18–3.70)	0.57 (0.26–1.25)
Indian sub-continent	3905	1844 (47.2)	414 (22.5)	1.21 (1.00–1.46)	0.96 (0.68–1.35)	0.64 (0.49–0.83)	0.64 (0.36–1.12)
Mixed/other	904	436 (48.2)	101 (23.2)	0.97 (0.78–1.21)	0.80 (0.54–1.20)	0.58 (0.44–0.78)	0.50 (0.27–0.93)
Missing	167	74 (44.3)	13 (17.6)				
**Time since entry to the UK**							
UK born	800	474 (59.3)	86 (18.1)	1.00	1.00	1.00	1.00
Within 2 years	999	480 (48.0)	107 (22.3)	1.75 (1.52–2.01)	1.51 (1.09–2.10)	2.06 (1.70–2.50)	2.56 (1.62–4.05)
2–5 years	1450	694 (47.9)	156 (22.5)	0.72 (0.61–0.84)	1.16 (0.85–1.59)	0.99 (0.81–1.22)	1.72 (1.10–2.70)
5–10 years	1200	588 (49.0)	134 (22.8)	0.87 (0.74–1.01)	1.20 (0.87–1.65)	1.16 (0.94–1.42)	1.83 (1.15–2.89)
More than 10 years	1692	869 (51.4)	185 (21.3)	0.95 (0.83–1.09)	1.15 (0.85–1.56)	1.28 (1.04–1.57)	1.42 (0.91–2.23)
Missing	812	383 (47.2)	76 (19.8)				
**TB lineage**							
Beijing	335	245 (73.1)	34 (13.9)	1.00	1.00	1.00	1.00
Euro-American	2007	1067 (53.2)	236 (22.1)	0.46 (0.39–0.54)	1.21 (0.79–1.87)	0.41 (0.34–0.49)	1.16 (0.70–1.93)
Central Asian Strain	2325	1337 (57.5)	255 (19.1)	0.75 (0.65–0.87)	1.36 (0.89–2.09)	0.76 (0.64–0.90)	1.37 (0.82–2.26)
East Asian Indian	1403	495 (35.3)	133 (26.9)	0.48 (0.41–0.58)	2.18 (1.40–3.42)	0.55 (0.45–0.67)	2.07 (1.23–3.48)
Other	881	342 (38.8)	85 (24.9)	0.66 (0.56–0.79)	2.17 (1.36–3.47)	0.62 (0.51–0.75)	1.92 (1.12–3.30)
Missing	2						
**IMD decile**							
1	1352	706 (52.2)	160 (22.7)	–	–	–	–
2	1407	747 (53.1)	156 (20.9)	–	–	–	–
3	1157	587 (50.7)	136 (23.2)	–	–	–	–
4	876	441 (50.3)	72 (16.3)	–	–	–	–
5	616	297 (48.2)	75 (25.3)	–	–	–	–
6	446	215 (48.2)	45 (20.9)	–	–	–	–
7	313	147 (47.0)	26 (17.7)	–	–	–	–
8	242	122 (50.4)	30 (24.6)	–	–	–	–
9	220	83 (37.7)	17 (20.5)	–	–	–	–
10	169	72 (42.6)	12 (16.7)	–	–	–	–
Missing	155	71 (45.8)	15 (21.1)	–	–	–	–
For each decile increase	–	–	–	0.93 (0.92–0.95)	1.03 (1.00–1.07)	0.97 (0.95–0.99)	1.03 (0.99–1.08)
**Drug misuse**							
No	6371	3180 (49.9)	675 (21.2)	1.00	1.00	1.00	1.00
Yes	63	44 (69.8)	7 (15.9)	0.41 (0.21–0.82)	0.34 (0.10–1.18)	0.49 (0.27–0.90)	0.31 (0.06–1.66)
Missing	519	264 (50.9)	62 (23.5)				
**Alcohol misuse**							
No	6221	3117 (50.1)	654 (21.0)	1.00	1.00	1.00	1.00
Yes	106	58 (54.7)	13 (22.4)	1.44 (1.13–1.83)	0.89 (0.47–1.66)	1.79 (1.34–2.38)	1.09 (0.47–2.51)
Missing	626	313 (50.0)	77 (24.6)				
**Homelessness**							
No	6373	3203 (50.3)	679 (21.2)	1.00	1.00	1.00	1.00
Yes	99	56 (56.6)	7 (12.5)	0.29 (0.12–0.72)	0.71 (0.21–2.33)	0.23 (0.09–0.58)	0.62 (0.10–3.94)
Missing	481	229 (47.6)	58 (25.3)				
**Imprisonment**							
No	6220	3119 (50.1)	657 (21.1)	1.00	1.00	1.00	1.00
Yes	96	61 (63.5)	8 (13.1)	0.06 (0.03–0.13)	1.11 (0.94–1.31)	0.17 (0.04–0.82)	0.36 (0.01–8.94)
Missing	637	308 (48.4)	79 (25.6)				

*IRR:* incidence rate ratio (Poisson part) for an increased number of subsequent clustered cases. *OR:* odds ratio (zero-inflated part) for the odds of being a non-clustered case, compared to being the first extra-pulmonary case of a cluster. *IMD:* index of multiple deprivation score. IMD score deciles represent relative levels of deprivation of income, employment, health, education, housing and services, crime and living environment for small areas in England and Wales, where 1 = most deprived and 10 = least deprived [29, 30]

^**≠**^Adjusted for all variables shown in the table. The multivariable model included 3576 extra-pulmonary TB cases after 633 were excluded due to missing data on one or more of sex (*n* = 3), ethnicity (*n* = 106), time since entry to the UK (*n* = 505), IMD score (*n* = 99) or TB lineage (*n* = 1)

^**†**^Cases missing data were considered not to have these social risk factors

In a sensitivity analysis, we examined the number of subsequent cases following the first pulmonary case in each cluster, rather than stratifying the analysis by the site of TB disease of the first patient in the cluster. This analysis showed results consistent with the main analysis (Additional file 1: Table S1).

#### Outcome 2: HIV and the likelihood of being a subsequent case in a cluster (a surrogate for recent TB infection)

TB cases with HIV co-infection were less likely to be a subsequent case in a cluster in univariable and multivariable analysis (multivariable OR 0.82 [0.69–0.98], Table 5), indicating that reactivation of LTBI was more likely to have been the source of disease for these individuals. A sensitivity analysis in which we assumed non-clustered cases and the first pulmonary case of each cluster (rather than the first case of the cluster irrespective of disease site) were the result of reactivation of LTBI and that all other clustered cases were the result of recent transmission showed consistent results (Additional file 1: Table S2).

**Table 5:** Univariable and multivariable logistic regression of factors associated with being a subsequent TB case in a cluster (a surrogate for recent infection) compared to being the first case or a non-clustered case, in England, Wales and Northern Ireland from 2010 to 2014

	Univariable OR (95% CI)	Multivariable^**≠**^ OR (95% CI)
**HIV status**		
Negative	1.00	1.00
Positive	0.85 (0.73–0.98)	0.82 (0.69–0.98)
**Year of TB notification**		
2010	1.00	1.00
2011	1.87 (1.70–2.07)	2.06 (1.84–2.31)
2012	2.60 (2.36–2.87)	3.06 (2.74–3.43)
2013	2.91 (2.63–3.22)	3.38 (3.02–3.80)
2014	2.74 (2.48–3.04)	3.17 (2.82–3.56)
**Sex**		
Female	1.00	
Male	1.10 (1.04–1.17)	1.09 (1.02–1.17)
**Age (years)**		
15–24	1.35 (1.24–1.47)	1.19 (1.08–1.32)
25–34	1.00	1.00
35–44	1.05 (0.96–1.14)	0.92 (0.83–1.02)
45–54	1.20 (1.09–1.32)	0.90 (0.80–1.01)
55–64	1.21 (1.07–1.35)	0.96 (0.83–1.10)
65+	0.71 (0.64–0.78)	0.51 (0.45–0.57)
**Ethnicity**		
White	1.00	1.00
Black African	1.03 (0.94–1.13)	1.51 (1.31–1.73)
Black Other	2.06 (1.72–2.47)	2.25 (1.82–2.78)
Indian sub-continent	0.72 (0.66–0.77)	0.92 (0.81–1.04)
Mixed/other	0.71 (0.65–0.79)	0.98 (0.85–1.13)
**Time since entry to the UK**		
UK born	1.00	1.00
Within 2 years	0.49 (0.44–0.54)	0.41 (0.36–0.47)
2–5 years	0.49 (0.44–0.53)	0.39 (0.35–0.44)
5–10 years	0.56 (0.51–0.62)	0.49 (0.43–0.55)
More than 10 years	0.65 (0.59–0.70)	0.61 (0.54–0.69)
**TB lineage**		
Beijing	1.00	1.00
Euro-American	0.47 (0.41–0.54)	0.38 (0.33–0.45)
Central Asian Strain	0.58 (0.50–0.66)	0.63 (0.54–0.74)
East Asian Indian	0.23 (0.19–0.26)	0.23 (0.19–0.28)
Other	0.37 (0.31–0.42)	0.32 (0.27–0.38)
**IMD decile**		
For each decile increase	0.97 (0.96–0.98)	0.98 (0.96–0.99)
**Drug misuse**		
No	1.00	1.00
Yes	2.62 (2.24–3.08)	1.53 (1.25–1.87)
**Alcohol misuse**		
No	1.00	1.00
Yes	1.65 (1.43–1.91)	1.21 (1.01–1.45)
**Homelessness**		
No	1.00	1.00
Yes	1.58 (1.35–1.84)	1.03 (0.85–1.24)
**Imprisonment**		
No	1.00	1.00
Yes	2.16 (1.84–2.54)	1.26 (1.03–1.54)

*OR:* odds ratio, *IMD:* index of multiple deprivation score

^**≠**^Adjusted for all variables shown in the table. The multivariable model included 16,171 TB cases after 2693 were excluded due to missing data on one or more of sex (*n* = 20), ethnicity (*n* = 470), time since entry to the UK (*n* = 2041), IMD score (*n* = 489) and/or TB lineage (*n* = 2)

^**†**^Cases missing data were considered not to have these social risk factors

## Discussion

In this retrospective cohort study undertaken in England, Wales and Northern Ireland, we found that pulmonary TB patients with HIV seemed to transmit disease less than individuals without this co-infection, i.e. they had fewer subsequent clustered cases than those without HIV. This is consistent with the results of contact studies across high- and low-burden settings, which have found lower risks of LTBI and TB disease among the contacts of HIV-positive patients than HIV-negative TB patients [3–6]. This adds weight to the suggestion that patients with pulmonary TB and HIV may be less infectious than individuals without HIV co-infection. Among EPTB cases, we found a strong association between HIV co-infection and not being the first case of a cluster, again suggesting that patients with HIV are substantially less infectious. However, where HIV-positive EPTB patients were the first case of a cluster, they had substantially more subsequent clustered cases than HIV-negative EPTB patients. As it is generally accepted that patients with only EPTB disease are not infectious, it is unlikely these patients are driving transmission within these larger clusters. Transmission may have occurred from undiagnosed patients or patients without a known strain type, with the HIV-positive EPTB case appearing to be the first case due to more rapid disease progression or earlier presentation to clinical services. Increased cluster size may also be the result of transmission chains within clusters. HIV prevalence was higher among subsequent cases in clusters with an HIV-positive first case than clusters with HIV-negative first cases; it is therefore likely that the increased cluster size is because HIV infection is concentrated within some communities, and so the contacts of the HIV-positive infectious case are more likely to be susceptible to infection and progression to active disease. There may also be other social factors influencing transmission which differ between clusters with respect to HIV status, for example, living conditions, social mixing patterns and health-seeking behaviours, which we were not able to account for in this study.

Regardless of whether these HIV-positive cases are the ‘true’ first case in a cluster or merely the first case in a cluster to develop symptoms or present to care, the first observable patient is still a point at which interventions to diagnose patients earlier or investigate clusters can be targeted. National Institute for Health and Care Excellence guidelines currently suggest contact tracing is unnecessary for EPTB cases, and this is supported by a recent cost-effectiveness study [31]. However, our findings demonstrate that whilst EPTB cases may not drive transmission, EPTB cases with HIV can be the first observable case of a substantially larger cluster, which is important for directing cluster investigations. Furthermore, as around 50% of co-infected patients are only diagnosed with HIV at the time of their TB diagnosis [32], targeting HIV screening and LTBI treatment to the contacts of TB patients with HIV could result in earlier diagnosis of HIV infections, providing the opportunity to initiate anti-retroviral therapy and prevent TB disease from occurring [33].

We found a negative association between HIV co-infection and being a subsequent case in a cluster, compared to being the first case or a non-clustered case. This suggests that TB in patients with HIV is more often the result of reactivation of remotely-acquired LTBI than recent infection. These TB cases may be preventable if PLHIV, particularly those born abroad, could be tested and treated for LTBI. This finding contrasts with that of a meta-analysis of the association between HIV and clustering of TB cases in HIV-endemic populations [34], and more recent studies using WGS [35, 36], which concluded that HIV-associated TB was more often the result of recent infection than reactivation of LTBI. This difference is likely the result of the different settings; the higher incidence of TB in the general population in countries where HIV is endemic will lead to a greater force of infection which may differentially affect immunocompromised PLHIV. In contrast, in the UK (and other low-burden settings), the majority of TB cases are in foreign-born patients and transmission is generally considered to be low [9]. As there is generally less exposure to TB, HIV contributes more to reactivation of LTBI than to new TB infections.

Our study benefits from a large sample of all culture-positive TB cases strain typed at ≥ 23 loci in England, Wales and Northern Ireland over a 5-year period and represents over 80% of culture-confirmed TB cases and over 50% of all TB cases in the country during this time. This coverage was comparable to national studies of a similar size in the Netherlands [18, 37] and considerably higher than the 31% coverage in a previous study in England which did not include data on HIV co-infection [10, 38]. Studies in Norway and Denmark have achieved higher rates of coverage nationally (67–69% of all TB cases); however, these studies had limited or no information on HIV status and much smaller overall sample sizes [39, 40]. The cases included in the analysis did not substantially differ in terms of age, sex, ethnicity, place of birth (UK or abroad), year of TB diagnosis or presence of social risk factors from those not included (data not shown).

24-loci MIRU-VNTR is a highly discriminative, high-throughput method of genotyping *MTBC* [41, 42] and has been widely used in TB cluster investigations. However, analyses using whole-genome sequencing (WGS) have demonstrated that indistinguishable 24-loci MIRU-VNTR profiles do not always have sufficiently high resolution to distinguish between closely related, but distinct, lineages [17, 43].

As of 2014, over 95% of adults (18–64 years) diagnosed with TB, who previously did not know their HIV status, were tested for HIV [44]. It is possible that a small number of individuals with undiagnosed HIV were mistakenly classified as HIV-negative. We would expect any such misclassification to either be non-differential or for HIV-positive people to be more likely to be tested. Any misclassification would therefore have biased our results towards the null, making the true effect of HIV infection greater than stated, and so we do not consider this a major limitation of our study.

We classed clustered TB cases as being the first case or a subsequent case in clusters according to their earliest date of evidence of TB. Consequently, we may have misclassified the order of patients within clusters, as patients may not develop symptoms or present to care in the order in which they were infected. In particular, TB patients diagnosed with HIV may be diagnosed sooner. If this is the case, we would expect differential misclassification of TB patients with HIV as the first case in a cluster, when in fact they may just be the first patient in that cluster who developed symptoms or presented to care. However, we found that HIV-positive cases typically had fewer subsequent cases and were less likely to be subsequent cases in clusters, and so any misclassification to this effect would have biased our results towards the null and caused underestimation of the impact of HIV. Furthermore, under 50% of TB patients are aware of their HIV infection when diagnosed with TB [32]; therefore, this would not have influenced the time it took them to present to care, although their disease may have progressed more quickly. We also, where possible (Additional file 1: Table S3), used symptom onset date to determine the order of patients in clusters, as much onward transmission will occur before a TB patient is diagnosed.

Shared strain types may not represent recent transmission, particularly in patients born abroad who may have been infected with common endemic strain types before entering the UK [9]. This could have caused us to overestimate the proportion of TB attributable to recent transmission. Conversely, cases which appeared to have a unique strain type could be the result of recent infection acquired outside of England, Wales and Northern Ireland. Whilst our sample size was large, we were only able to include approximately 50% of TB cases nationally in our analysis as strain typing relies on culture of mycobacterial samples. Low sampling fractions result in underestimation of the extent of clustering [45, 46], as cases can be misclassified as not-clustered if the case they cluster with has not been strain typed. However, it has been shown that a low sampling fraction does not bias estimations of risk factors associated with clustering [45, 46].

We chose not to include data on the CD4 count of HIV-positive individuals. Due to the retrospective nature of our study, which used routinely collected data, it was not possible to determine when TB transmission occurred. We therefore were unable to determine the CD4 count of HIV-positive individuals at the time of transmission and so were unable to explore any possible association between CD4 count and propensity to transmit TB. We were also unable to include data on other factors that may have been relevant, such as socioeconomic status and diabetes, as these data were not routinely recorded.

Data on HIV status was not available for children, and therefore children could not be included in this analysis. Children are also less likely to have sputum samples taken and therefore less likely to be strain-typed. To limit bias, we included children when determining whether TB cases were clustered and whether a case was the first or a subsequent case in a cluster and then excluded patients aged < 15 years from the risk factor analysis. TB in children living with HIV is relatively rare in the UK [47], and children with TB are considered unlikely to transmit TB; therefore, the impact of HIV on TB transmission from children is likely to be minimal.

## Conclusions

In conclusion, we report that pulmonary TB patients with HIV had fewer subsequent clustered cases than patients without HIV. However, when patients with HIV and EPTB were the first case of a cluster, they had a higher number of subsequent cases. HIV prevalence was higher among the subsequent cases of HIV-positive first cases than the subsequent cases of HIV-negative first cases, suggesting that the higher number of subsequent cases for EPTB patients with HIV could be because their contacts are more susceptible to infection and progression of disease. Similarly, EPTB patients with HIV may be a sentinel marker for other factors driving recent transmission, and contact tracing should not be discounted for these cases. Our findings suggest that screening the contacts of TB patients with HIV for both HIV and LTBI could be considered. Furthermore, TB cases with HIV were less likely to be a subsequent case within a cluster, which suggests that HIV-associated TB is more often due to reactivation of LTBI rather than recent infection. More widespread testing for LTBI and preventive therapy among people living with HIV could decrease the incidence of HIV-associated TB.

## Supplementary Information

**Additional file 1: Table S1:** Sensitivity analysis for a multivariable zero-inflated Poisson regression of factors associated with the number of subsequent clustered cases for the first pulmonary TB case in each cluster in England, Wales and Northern Ireland, 2010–2014. **Table S2:** Sensitivity analysis for a multivariable logistic regression of factors associated with being a subsequent TB case in a cluster (a surrogate for recent infection) compared to being the first pulmonary case or a non-clustered case, in England, Wales and Northern Ireland from 2010 to 2014. **Table S3:** The date used to determine the position of a case in a cluster for the 18,864 cases included in the analysis.

## Data Availability

Aggregate data that support the findings of this study are available on reasonable request from PHE. The individual level data are not publicly available, as the data were collected in adherence with the legal framework governing use of confidential personally identifiable information.

## Abbreviations

EPTB: Extra-pulmonary tuberculosis
ETS: Enhanced tuberculosis surveillance
HIV: Human immunodeficiency virus
IRR: Incidence rate ratio
LTBI: Latent tuberculosis infection
MIRU-VNTR: Mycobacterial interspersed repetitive units–variable number tandem repeats
*MTBC*: *Mycobacterium tuberculosis complex*
OR: Odds ratio
PHE: Public Health England
TB: Tuberculosis
TST: Tuberculin skin test
