# Supplementary material for: The impact of HIV infection on tuberculosis transmission in a country with low tuberculosis incidence: a national retrospective study using molecular epidemiology
Source: BMC Med. 2020 Dec 14;18:385. doi: 10.1186/s12916-020-01849-7 (PMC7734856; doi:10.1186/s12916-020-01849-7)
Supplement: Supplementary file 1 — Additional file 1: Table S1: Sensitivity analysis for a multivariable zero-inflated Poisson regression of factors associated with the number of subsequent clustered cases for the first pulmonary TB case in each cluster in England, Wales and Northern Ireland, 2010–2014. Table S2: Sensitivity analysis for a multivariable logistic regression of factors associated with being a subsequent TB case in a cluster (a surrogate for recent infection) compared to being the first pulmonary case or a non-clustered case, in England, Wales and Northern Ireland from 2010 to 2014. Table S3: The date used to determine the position of a case in a cluster for the 18,864 cases included in the analysis. [file 12916_2020_1849_MOESM1_ESM.docx]

## Supplementary Tables

Table S1: Sensitivity analysis for a multivariable zero-inflated Poisson regression of factors associated with the number of subsequent clustered cases for the first pulmonary TB case in each cluster in England, Wales and Northern Ireland, 2010-2014

|  | **Multivariable≠**  **(Number of subsequent cases)** | **Multivariable≠**  **(Non-clustered case)** |
| --- | --- | --- |
|  | **IRR (95% CI)** | **OR (95% CI)** |
| **HIV status** |  |  |
| Negative | 1.00 | 1.00 |
| Positive | 0.76 (0.66-0.87) | 1.11 (0.80-1.54) |
| **Year of TB diagnosis** |  |  |
| 2010 | 1.00 | 1.00 |
| 2011 | 0.64 (0.60-0.68) | 1.53 (1.30-1.81) |
| 2012 | 0.38 (0.34-0.43) | 1.54 (1.25-1.89) |
| 2013 | 0.40 (0.34-0.47) | 2.38 (1.83-3.10) |
| 2014 | 0.59 (0.47-0.74) | 4.04 (2.87-5.70) |
| **Sex** |  |  |
| Female | 1.00 | 1.00 |
| Male | 1.00 (0.95-1.06) | 0.81 (0.70-0.93) |
| Missing |  |  |
| **Age (years)** |  |  |
| 15-24 | 0.82 (0.75-0.89) | 0.77 (0.62-0.94) |
| 25-34 | 1.00 | 1.00 |
| 35-44 | 1.31 (1.22-1.41) | 0.99 (0.81-1.22) |
| 45-54 | 0.94 (0.85-1.04) | 1.06 (0.83-1.36) |
| 55-64 | 0.99 (0.89-1.11) | 1.35 (1.01-1.81) |
| 65+ | 1.01 (0.91-1.11) | 1.96 (1.53-2.52) |
| **Ethnicity** |  |  |
| White | 1.00 | 1.00 |
| Black African | 1.21 (1.10-1.33) | 0.90 (0.69-1.18) |
| Black Other | 0.86 (0.74-1.01) | 0.58 (0.37-0.92) |
| Indian sub-continent | 0.93 (0.85-1.01) | 1.19 (0.94-1.51) |
| Mixed/other | 0.73 (0.65-0.82) | 1.13 (0.85-1.49) |
| Missing |  |  |
| **Time since entry to the UK** |  |  |
| UK born | 1.00 | 1.00 |
| Within 2 years | 0.64 (0.59-0.71) | 1.27 (0.99-1.62) |
| 2-5 years | 0.78 (0.71-0.86) | 1.36 (1.06-1.75) |
| 5-10 years | 0.73 (0.67-0.80) | 1.24 (0.96-1.59) |
| More than 10 years | 0.84 (0.77-0.91) | 1.10 (0.87-1.40) |
| Missing |  |  |
| **TB lineage** |  |  |
| Beijing | 1.00 | 1.00 |
| Euro-American | 0.46 (0.42-0.51) | 1.11 (0.80-1.55) |
| Central Asian Strain (CAS) | 0.78 (0.70-0.86) | 1.01 (0.72-1.43) |
| East Asian Indian (EAI) | 0.52 (0.46-0.60) | 1.55 (1.07-2.24) |
| Other | 0.44 (0.39-0.49) | 1.19 (0.83-1.71) |
| Missing |  |  |
| **IMD decile** |  |  |
| 1 | - | - |
| 2 | - | - |
| 3 | - | - |
| 4 | - | - |
| 5 | - | - |
| 6 | - | - |
| 7 | - | - |
| 8 | - | - |
| 9 | - | - |
| 10 | - | - |
| Missing | - | - |
| For each decile increase | 0.96 (0.95-0.97) | 1.00 (0.97-1.03) |
| **Drug misuse** |  |  |
| No | 1.00 | 1.00 |
| Yes | 0.87 (0.76-1.00) | 0.85 (0.56-1.29) |
| Missing |  |  |
| **Alcohol misuse** |  |  |
| No | 1.00 | 1.00 |
| Yes | 1.70 (1.54-1.87) | 1.18 (0.85-1.66) |
| Missing |  |  |
| **Homelessness** |  |  |
| No | 1.00 | 1.00 |
| Yes | 0.62 (0.54-0.72) | 0.88 (0.59-1.29) |
| Missing |  |  |
| **Imprisonment** |  |  |
| No | 1.00 | 1.00 |
| Yes | 1.12 (0.98-1.27) | 0.83 (0.56-1.23) |
| Missing |  |  |
| **Smear status** |  |  |
| Smear positive | 1.00 | 1.00 |
| Smear negative or unknown | 0.85 (0.81-0.90) | 1.17 (1.02-1.34) |

**IRR**: incidence rate ratio (Poisson part) for an increased number of subsequent clustered cases. **OR**: odds ratio (zero-inflated part) for the odds of being a non-clustered case, compared to being the first extra-pulmonary case of a cluster. **IMD**: index of multiple deprivation score.
This sensitivity analysis analysed the number of subsequent cases for the first pulmonary case in each cluster (regardless of whether that was the first case in a cluster or a later case), rather than the first case in each cluster irrespective of disease site (as in the main analysis).

Table S2: Sensitivity analysis for a multivariable logistic regression of factors associated with being a subsequent TB case in a cluster (a surrogate for recent infection) compared to being the first pulmonary case or a non-clustered case, in England, Wales and Northern Ireland from 2010-2014

|  | **Multivariable≠ OR (95% CI)** |
| --- | --- |
| **HIV status** |  |
| Negative | 1.00 |
| Positive | 0.70 (0.59-0.84) |
| **Year of TB notification** |  |
| 2010 | 1.00 |
| 2011 | 1.62 (1.45-1.80) |
| 2012 | 2.10 (1.88-2.33) |
| 2013 | 2.23 (2.00-2.49) |
| 2014 | 1.94 (1.73-2.17) |
| **Sex** |  |
| Female |  |
| Male | 1.07 (1.00-1.15) |
| **Age (years)** |  |
| 15-24 | 1.10 (1.00-1.22) |
| 25-34 | 1.00 |
| 35-44 | 0.92 (0.83-1.01) |
| 45-54 | 0.91 (0.81-1.02) |
| 55-64 | 0.96 (0.84-1.10) |
| 65+ | 0.51 (0.45-0.57) |
| **Ethnicity** |  |
| White | 1.00 |
| Black African | 1.64 (1.43-1.87) |
| Black Other | 2.21 (1.80-2.72) |
| Indian sub-continent | 1.00 (0.89-1.13) |
| Mixed/other | 1.03 (0.90-1.19) |
| **Time since entry to the UK** |  |
| UK born | 1.00 |
| Within 2 years | 0.43 (0.38-0.49) |
| 2-5 years | 0.45 (0.40-0.51) |
| 5-10 years | 0.53 (0.47-0.60) |
| More than 10 years | 0.64 (0.57-0.72) |
| **TB lineage** |  |
| Beijing | 1.00 |
| Euro-American | 0.39 (0.34-0.46) |
| Central Asian Strain (CAS) | 0.61 (0.52-0.72) |
| East Asian Indian (EAI) | 0.24 (0.20-0.29) |
| Other | 0.33 (0.28-0.39) |
| **IMD decile** |  |
| For each decile increase | 0.98 (0.96-0.99) |
| **Drug misuse** |  |
| No | 1.00 |
| Yes | 1.61 (1.32-1.96) |
| **Alcohol misuse** |  |
| No | 1.00 |
| Yes | 1.20 (1.00-1.43) |
| **Homelessness** |  |
| No | 1.00 |
| Yes | 0.97 (0.81-1.17) |
| **Imprisonment** |  |
| No | 1.00 |
| Yes | 1.16 (0.95-1.41) |

**IMD**: index of multiple deprivation. **OR**: odds ratio.

Table S3: The date used to determine the position of a case in a cluster for the 18,864 cases included in the analysis

| **Date used** | **Number** | **Percentage** |
| --- | --- | --- |
| Symptom onset date | 14,367 | 76.2 |
| Date of presentation | 480 | 2.5 |
| Specimen date | 3,134 | 16.6 |
| Date of diagnosis | 267 | 1.4 |
| Date of treatment start | 508 | 2.7 |
| Case report date | 108 | 0.6 |
